# Computational Imaging Method for Thermal Infrared Hyperspectral Imaging Based on a Snapshot Divided-Aperture System

**DOI:** 10.3390/s26061982

**Published:** 2026-03-22

**Authors:** Tianzhen Ma, Zhijing He, Bin Wu, Yutian Lei, Yijie Wang, Xinze Liu, Bingmei Guo, Jiawei Lu, Bo Cheng, Shikai Zan, Chunlai Li, Liyin Yuan

**Affiliations:** 1 Shanghai Institute of Technical Physics, Chinese Academy of Sciences, Shanghai 200083, China; matianzhen20@mails.ucas.ac.cn (T.M.); hezhijing21@mails.ucas.ac.cn (Z.H.); leiyutian24@mails.ucas.ac.cn (Y.L.); wangyijie22@mails.ucas.ac.cn (Y.W.); liuxinze@mail.sitp.ac.cn (X.L.); meiableyeah@163.com (B.G.); lujiawei@mail.sitp.ac.cn (J.L.); chengbo21@mails.ucas.ac.cn (B.C.); 2 University of Chinese Academy of Sciences, Beijing 100049, China; 3 College of Instrumentation & Electrical Engineering, Jilin University, Changchun 130061, China; wubin22@mails.jlu.edu.cn (B.W.); zansk23@mails.jlu.edu.cn (S.Z.)

**Keywords:** thermal infrared, computational imaging, hyperspectral imaging

## Abstract

**Highlights:**

**What are the main findings?**

- A divided-aperture snapshot thermal infrared multispectral camera is developed, enabling single-exposure acquisition of both thermal images and multispectral data, with precise sub-channel image registration achieved via a star-point array calibration method.
- A neural network-based computational imaging method is proposed, successfully reconstructing 127-channel hyperspectral data from only 9-channel low-dimensional multispectral measurements.

**What is the implication of the main findings?**

- This method achieves reconstruction from a multispectral to hyperspectral data cube while preserving system compactness and snapshot capability, offering a potential tool for hyperspectral sensing in fields such as environmental monitoring and industrial inspection.

**Abstract:**

To address the technical challenge of simultaneously achieving snapshot imaging capability and high spectral resolution in thermal infrared spectral imaging, this paper proposes a computational imaging method based on a snapshot divided-aperture imaging system. In this method, a self-developed divided-aperture snapshot multispectral camera is utilized to simultaneously capture nine low-spectral-resolution images in a single exposure. The precise registration of the sub-channel images is accomplished via a star-point array calibration method. To construct the spectral reconstruction dataset, a Fourier-transform infrared hyperspectral camera (FTIR HCam) is employed to simultaneously acquire hyperspectral data from real-world scenes. Based on this, a neural network model is applied to reconstruct 127-channel hyperspectral information from the low-dimensional multispectral measurements. Experimental results demonstrate that the proposed method effectively achieves hyperspectral reconstruction while maintaining system compactness and snapshot imaging capability, thus providing a viable technical approach for hyperspectral sensing in dynamic thermal infrared scenarios.

## 1. Introduction

Thermal infrared imaging technology is widely used in environmental monitoring [1,2], military reconnaissance [3,4], industrial inspection [5,6], and medical diagnosis [7,8,9] due to its unique advantages such as night-time observation, smoke penetration, and temperature sensing. However, with the growing complexity of observational demands, conventional thermal infrared imaging has shown limitations in the information dimension for applications such as material identification and camouflage detection, often requiring the support of fine spectral features provided by hyperspectral imaging. Meanwhile, research into dynamic processes such as gas diffusion [10,11,12], exhaust emissions [13,14,15], and combustion [16] imposes higher requirements on the temporal response capability of imaging systems, necessitating snapshot imaging capabilities [17]. Therefore, achieving the effective integration of hyperspectral imaging and snapshot imaging in the thermal infrared band has emerged as a significant research direction. Currently, traditional thermal infrared imaging systems often face trade-offs between snapshot imaging capability, spectral resolution, and system complexity, making it challenging to balance multiple performance aspects. This has spurred exploration into novel imaging architectures. In this context, developing a thermal infrared camera that combines snapshot imaging and hyperspectral information acquisition capabilities holds considerable importance. Such technology has the potential to enable both instantaneous scene capture and spectral analysis, thereby significantly enhancing the perception and identification of multi-dimensional information in complex environments.

Currently, common thermal infrared spectral imaging technologies [18,19] are primarily implemented through typical systems such as dispersive scanning, filter-based, or Fourier-transform approaches. However, when faced with the demand for snapshot hyperspectral imaging, these schemes exhibit significant limitations. Dispersive scanning systems often rely on scanning-based imaging, resulting in low temporal resolution and difficulty in effectively capturing rapid dynamic processes. Filter-based systems feature relatively simple structures, but they suffer from a limited number of spectral channels or restricted tuning speeds, making it challenging to achieve both high spectral resolution and snapshot imaging capability simultaneously. Fourier-transform imaging systems, based on interference principles, can achieve high spectral resolution, but they are characterized by structural complexity, susceptibility to environmental vibrations, and lengthy data acquisition and processing times. These common constraints collectively hinder the application of the aforementioned technologies in scenarios requiring both snapshot acquisition and high spectral resolution. Among various filter-based imaging schemes, divided-aperture spectral imaging systems [20,21,22] offer a relatively simple structure and enable parallel acquisition of multiple spectral channels, thereby possessing snapshot imaging capability. However, such systems typically support only a limited number of spectral channels, restricting their spectral resolution capability. Nevertheless, divided-aperture systems preserve snapshot spectral imaging characteristics while maintaining low complexity, providing a feasible foundation for addressing the challenge of balancing snapshot capability, system complexity, and spectral resolution. Further optimization of this technology is expected to advance toward higher spectral resolution while retaining system simplicity and snapshot capability.

With the development of computational imaging techniques [23,24], it has become feasible to reconstruct hyperspectral data cubes using snapshot divided-aperture imaging systems that are structurally simple and limited in spectral channels. The core principle lies in leveraging neural network algorithms integrated with prior spectral information to achieve hyperspectral information reconstruction under hardware constraints [25,26]. Following this technical pathway, this study designed and constructed a nine-channel divided-aperture snapshot multispectral camera (9-DAS MCam). To acquire the training data required, the system was synchronized with a commercial FTIR HCam, (model Hyper-Cam LW, Telops Inc., Quebec City, QC, Canada) for the simultaneous data acquisition of the same scene. Ground-truth hyperspectral data provided by the FTIR HCam were used to build a training dataset, enabling the neural network to learn the mapping relationship from multispectral to hyperspectral information, thus achieving spectral reconstruction from low-dimensional to high-dimensional representation. This paper focuses on detailing the optical system design and implementation of the 9-DAS MCam, the multi-channel image registration method based on a star-point array, the dataset construction pipeline, and the neural network-based spectral reconstruction algorithm. This work offers a viable technical pathway for developing compact thermal infrared spectral systems that integrate both high spectral resolution and snapshot imaging capability.

## 2. Methods

### 2.1. Design of the 9-DAS MCam

**Figure 1:**
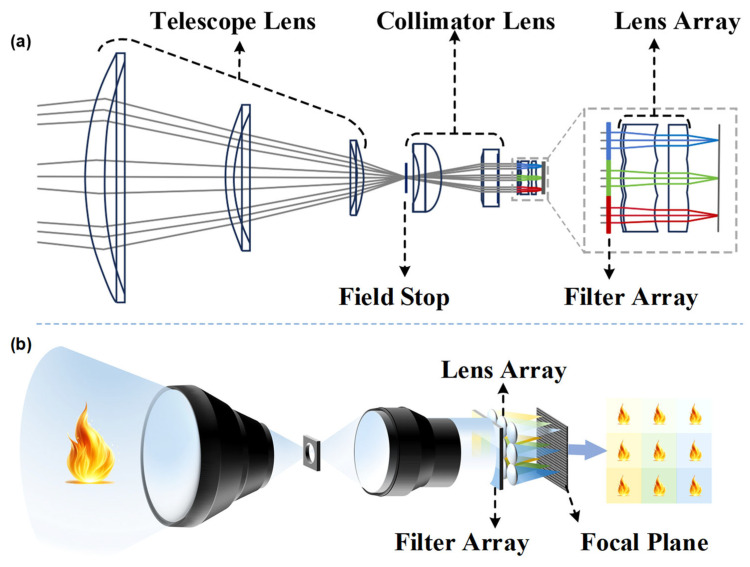
Principle and system composition of the 9-DAS MCam. (**a**) Schematic diagram of the optical path design. (**b**) Schematic diagram of the divided-aperture imaging principle, different colors represent different spectral bands.

To achieve snapshot spectral imaging, a 9-DAS MCam was developed in this study. The camera employs a divided-aperture optical imaging method (Figure 1a), whose core principle is as follows: by arranging an array of filters with different spectral bands and a corresponding lens array, the incident radiation captured in a single exposure is spatially partitioned, and the spectral information from each sub-aperture is modulated in parallel. This design enables a single focal plane array (FPA) detector to simultaneously record sub-images carrying distinct spectral information. After subsequent channel registration processing, a complete three-dimensional spectral data cube is ultimately constructed.

Specifically, the spectral splitting principle of the 9-DAS MCam is illustrated in Figure 1b. The thermal infrared radiation from the scene is first collected and converged by the telescope lens. It then passes through a field stop to define the imaging area and suppress stray light. Subsequently, the beam is converted into collimated light by the collimator lens, ensuring that it enters the subsequent spectral splitting assembly at an approximately normal incidence.

**Figure 2:**
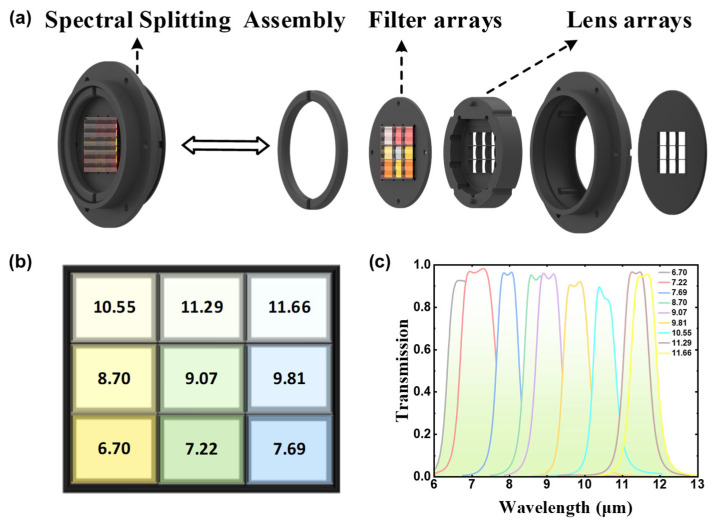
Spectral splitting assembly of the 9-DAS MCam. (**a**) Structural decomposition diagram of the spectral splitting assembly; (**b**) Layout of the 3 × 3 filter array and the central wavelength of each filter, where different colors represent filters with different transmission spectral bands; (**c**) Spectral transmittance curves of each filter.

As shown in Figure 2a, the spectral splitting assembly primarily consists of a customized 3 × 3 bandpass filter array paired with an aligned 3 × 3 lens array. Each element of the filter array (Figure 2b) is a bandpass filter with a designated transmission band. The transmission curves of these filters are meticulously designed so that within the target spectral range of 6–12 μm, the nine channel spectra are adjacent and partially overlapping (Figure 2c), effectively segmenting the continuous infrared spectrum into nine parallel sub-bands.

After being modulated by the filter array, the beams carrying different spectral band information are directed toward a precisely aligned lens array positioned behind it. Each lens element corresponds one-to-one with the filter unit in front of it and serves to converge the bandpass light of each channel onto pre-assigned corresponding regions of the FPA detector. Ultimately, these nine light spots, each carrying distinct spectral information, are simultaneously are captured simultaneously by a detector. Through this design, the system achieves snapshot imaging across nine parallel spectral channels within a single exposure. (Figure 1b).

### 2.2. Divided-Aperture Image Registration Method

The 9-DAS MCam operates in a snapshot mode characterized by “single exposure, parallel multispectral acquisition.” This hardware-level design inherently avoids the motion artifacts and temporal latency introduced by scanning, time-division, or transformation modulation in traditional spectral imaging [17], thereby providing a foundation for instantaneous spectral capture of dynamic scenes. However, the camera’s direct output consists of nine spatially tiled sub-images. To form a multispectral data cube suitable for spectral reconstruction, these sub-images must undergo precise inter-channel registration. This necessity arises mainly from optical machining and assembly tolerances, as well as subtle distortion differences among the individual sub-aperture imaging paths. These factors cause pixel-level positional shifts in the same object point across the nine sub-images, preventing direct alignment of the channel images. Therefore, prior to spectral reconstruction, sub-channel image registration must be performed to correct spatial mismatches between channels and achieve pixel-level spectral alignment. This step is an essential preprocessing procedure for ensuring subsequent high-fidelity spectral reconstruction.

To address the spatial geometric misalignment among the channels of the 9-DAS MCam, feature-based image matching methods often suffer from insufficient accuracy and reliability when dealing with low-quality thermal infrared images or scenes lacking distinctive features. Therefore, this study adopts an image registration method that integrates hardware calibration with algorithmic processing [27], replacing approaches that rely solely on post-processing. The core of this method involves using a star-point array to generate regularly distributed artificial feature points on the image plane. Through hardware measurement and centroid extraction, the positional offsets between channels are obtained, and a geometric transformation model is constructed to achieve stable registration with “one-time calibration, long-term validity.” The entire registration process primarily consists of two key steps: channel deviation measurement and image geometric transformation.

#### 2.2.1. Star-Point Array-Based Measurement of Inter-Channel Deviation

**Figure 3:**
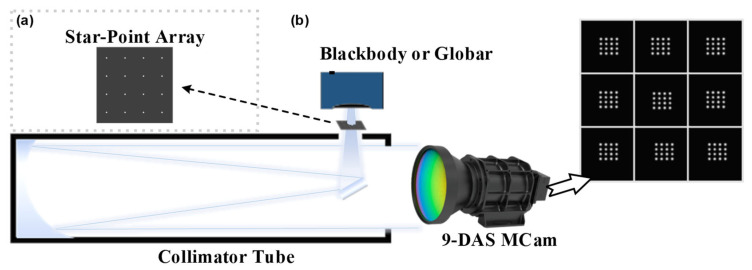
Star-point array calibration method for channel registration of the 9-DAS MCam. (**a**) The star-point array used for calibration; (**b**) Schematic diagram of the calibration for multi-channel image geometric deviation measurement based on the star-point array.

To accurately measure the relative positional deviations among the imaging channels of the 9-DAS MCam, this study designed a specialized star-point array calibration plate, the structure of which is shown in Figure 3a. This calibration plate consists of a regularly arranged array of micro-apertures fabricated on an opaque substrate, with each aperture referred to as a “star point.” Under illumination by a thermal infrared source, each star point forms a diffraction blur spot on the detector’s imaging plane. By applying a centroid extraction algorithm, the precise image-plane coordinates of each star point within its corresponding channel image can be calculated. To simultaneously ensure pixel accuracy in centroid localization and the separability of star point images, two key constraints must be satisfied: the energy of the diffraction blur spot generated by a single star point must be concentrated within approximately 3 × 3 detector pixels to guarantee centroid localization accuracy; simultaneously, the imaging separation between adjacent star points on the detector should be no less than 8 pixels to avoid misidentification of connected regions during image processing. Therefore, the star point diameter d requires rigorous design based on specific parameters of the optical system, and its calculation formula is as follows:

(1) $$d=\frac{n\cdot a\cdot f^{\prime }}{f}$$

Here, n represents the ratio between the ideal image size of the star point and the detector pixel pitch, a denotes the detector pixel pitch, $f^{\prime }$ is the focal length of the collimator tube, and f is the focal length of the 9-DAS MCam. In order to achieve pixel calibration accuracy, the value n=1.5 is adopted in the design to ensure that the blur spot size meets the aforementioned requirements.

**Figure 4:**
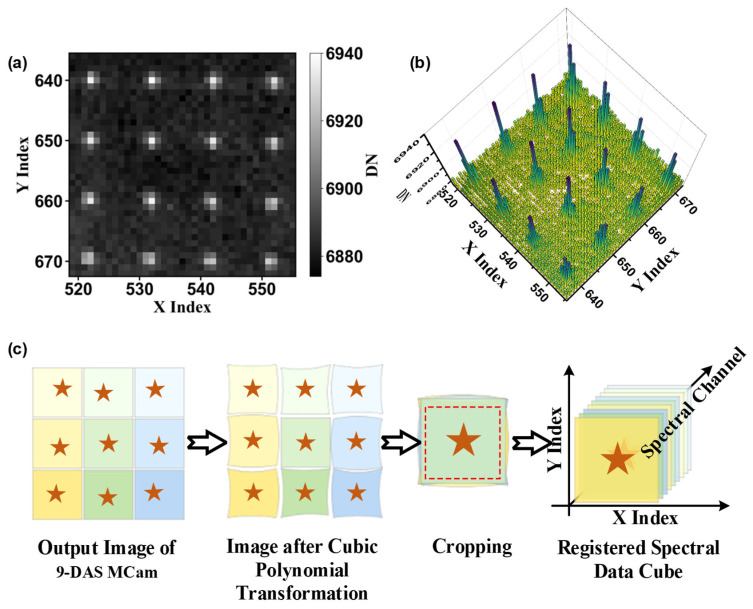
Imaging of the star-point array and channel registration workflow. (**a**) Imaging result of the star-point array within a single sub-channel; (**b**) Grayscale distribution of the star-point array blur spots on the detector; (**c**) Schematic diagram of the multi-channel registration process.

As shown in Figure 3b, during the measurement process, the star-point array calibration plate is positioned at the focal plane of the collimator tube and uniformly illuminated by a thermal infrared source, such as a blackbody or a globar. The resulting collimated parallel beam enters the optical system of the 9-DAS MCam, ultimately forming a composite image consisting of nine independent star-point arrays on the detector—with each imaging channel corresponding to a complete star-point array, as illustrated in Figure 4a. To cover the entire detector plane and obtain sufficient calibration point data, the 9-DAS MCam is moved via a motorized translation stage, and the acquisition process described above is repeated at different field positions until the star-point images uniformly cover the entire image plane. Figure 4b shows the typical intensity distribution of the star-point array blur spots on the detector. Based on this distribution, the gray-weighted centroid method is employed to calculate the centroid coordinates $\left(\hat{x},\hat{y}\right)$ of each star point image, using the following formula:

(2) $$\hat{x}=\frac{\sum_{i=1}^{n}x_{i}I_{i}}{\sum_{i=1}^{n}I_{i}},\;\;\hat{y}=\frac{\sum_{i=1}^{n}y_{i}I_{i}}{\sum_{i=1}^{n}I_{i}}$$

where $\left(x_{i},y_{i}\right)$ denotes the pixel coordinates covering the energy distribution of the blur spot, and $I_{i}$ represents the corresponding grayscale value of each pixel. The summation is performed over all n effective pixels encompassed by the energy distribution of the blur spot. After imaging the star-point array across the entire field of view (FOV) and computing the centroids of the star points, the positional deviation matrix between channels can be obtained by comparing the centroid positions of corresponding star points in the edge channels with those in the central channel. This matrix provides the data foundation for constructing the inter-channel geometric transformation model in subsequent steps.

#### 2.2.2. Image Geometric Transformation

Image registration typically involves three steps: feature detection, feature matching, and geometric transformation [28,29,30]. In the star-point array-based registration method employed in this study, the deviations among the nine channels have been pre-acquired through artificially arranged feature points during the calibration stage. This allows the method to circumvent the conventional processes of feature detection and matching, enabling the direct establishment and application of a geometric transformation model.

To minimize geometric misalignment among divided-aperture subchannels, based on the deviation of corresponding points between edge channels and the central channel, a geometric transformation is applied to the coordinates of corresponding points in the edge channels using a two-dimensional cubic polynomial. The polynomial coefficients are calculated by the least squares method, thereby minimizing the coordinate mapping deviation globally. Taking the central channel image as the reference coordinate system, let  (x,y) be the pixel coordinates in the reference channel and (x,y) be the corresponding coordinates in the channel to be corrected. The transformation relationship adopted in this study is expressed by a cubic polynomial as follows:

(3) $$x^{\prime }=\sum_{i=0}^{3}\sum_{j=0}^{3-i}a_{ij}x^{i}y^{j},\;\;y^{\prime }=\sum_{i=0}^{3}\sum_{j=0}^{3-i}b_{ij}x^{i}y^{j}$$

where $\left\{a_{ij},b_{ij}\right\}$ are the polynomial coefficients, determined by fitting the deviation matrix data mentioned earlier. When applying the above geometric transformation for coordinate mapping, to preserve the continuity of image intensity variation and restore the original information as fully as possible, this study employs the bilinear interpolation method to resample the transformed edge channel images, thereby achieving a smooth transition of pixel values.

Based on the eight transformed edge channel images and the center channel image, masks are generated. The effective overlapping regions of the nine channels are obtained through logical operations, followed by extracting the maximum inscribed rectangle of this common area. According to the boundaries of this rectangle, all channel images are uniformly cropped, thereby removing invalid edge pixels caused by geometric transformations and parallax.

In the final multispectral data cube, the pixel coordinate grid is strictly aligned with the physical pixel grid of the central channel. Throughout the registration process, the center channel serves as the absolute reference and maintains its original geometric state. Data from all other edge channels are mapped onto this reference grid via polynomial transformation. The subsequent cropping step merely extracts a unified, valid common region from each channel, which is effectively a translation of the logical origin and does not alter the definition of the final physical coordinate system.

Figure 4c illustrates the processing flow for image registration between the divided-aperture sub-channels. A hardware calibration process using a star-point array is employed to extract the deviation field between the edge channels and the central channel, which is then used to fit a geometric transformation model. This approach effectively mitigates the inherent inter-channel FOV discrepancies and optical distortions of the divided-aperture imaging system, ultimately outputting pixel-aligned nine-channel spectral images. This provides a reliable registration data foundation for the subsequent reconstruction from multispectral to hyperspectral data.

### 2.3. Dataset Construction

To achieve spectral reconstruction from the 9-DAS MCam multispectral data to hyperspectral data, constructing a paired dataset of “multispectral input–hyperspectral label” is crucial. The input data consist of the 9-channel multispectral data cube acquired by the 9-DAS MCam and registered following the procedure described in Section 2.2, from which the multispectral grayscale vector for each pixel is extracted. The label data correspond to the hyperspectral radiance vectors of the same pixels obtained at the same moment from the hyperspectral data cube captured by the commercial FTIR HCam, serving as the ground truth for spectral reconstruction. This dataset is used to train the subsequent neural network model; therefore, strict pixel-level alignment between the input and label data is essential.

**Figure 5:**
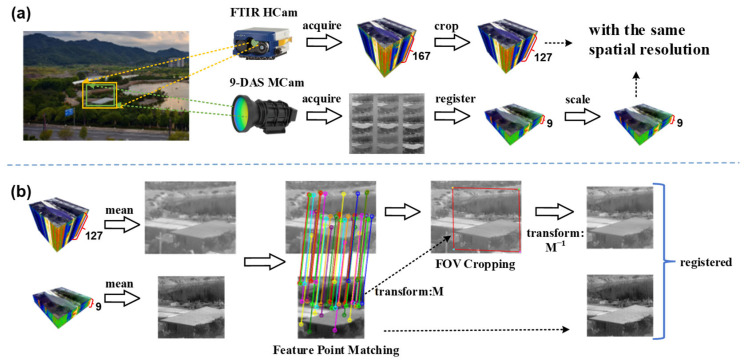
Data acquisition and registration workflow. (**a**) Data acquisition and spatial resolution unification; (**b**) Dual camera image registration pipeline. The spectral data cubes in this figure are visualized through a false-color composite.

The data acquisition follows a dual-camera synchronous observation scheme, with the specific arrangement illustrated in Figure 5a. The two cameras are positioned adjacent to each other and simultaneously image the same distant scene. However, due to inherent differences in optical design, detector specifications, and mounting positions, the captured images exhibit inconsistencies in ground sampling distance, incomplete overlap of the FOV, and varying optical distortion patterns. These issues prevent the direct establishment of pixel-level correspondence between the two sets of images. Therefore, a dedicated registration pipeline must be applied to correct them. This procedure begins with image resampling to unify the spatial resolution. Subsequently, spatial discrepancies are corrected through feature-point matching, FOV cropping, and geometric transformation, ultimately achieving pixel-level registration.

During the acquisition process, the spectral resolution of the FTIR HCam was set to 4 cm^−1^, covering a spectral range of 7.7–12.6 μm, resulting in the acquisition of 167 spectral bands. Due to the degradation in detector signal-to-noise ratio (SNR) towards the spectral response edges, the raw data cube was spectrally cropped. Only the central 127 channels within the 8–12 μm range, which exhibit relatively higher SNR, were retained as the final hyperspectral label data for neural network training.

#### 2.3.1. Unification of Spatial Resolution

To address the inconsistency in spatial resolution between the 9-DAS MCam and the FTIR HCam—since spatial resolution is determined by the instantaneous field of view (IFOV)—this study adopts a spatial resampling method based on IFOV matching to unify their spatial resolutions, as illustrated in Figure 5a. For the 9-DAS MCam, its $IFOV_{1}$, is determined by the pixel size  a and the focal length  f:

(4) $$IFOV_{1}=\frac{a}{f}$$

For the FTIR HCam, the $IFOV_{2}$, can be calculated from its FOV and the number of pixels n in the corresponding direction:

(5) $$IFOV_{2}=\frac{FOV}{n}$$

To achieve spatial resolution uniformity between the 9-channel multispectral data cube of the 9-DAS MCam and the 127-channel hyperspectral data cube of the FTIR HCam, the scaling factor ρ is defined as the ratio of their IFOV:

(6) $$\rho =\frac{IFOV_{1}}{IFOV_{2}}$$

That is, while keeping the FOV unchanged, the spatial dimensions of the 9-DAS MCam image need to be scaled by a factor of ρ relative to the original image. The scaling process employs bilinear interpolation for resampling to ensure a smooth transition of spectral features during resampling. Through the above processing, the data cubes acquired by the two cameras achieve unified spatial resolution.

#### 2.3.2. Feature Point-Based Image Registration

After spatial resolution unification, spatial offsets caused by differences in camera mounting positions, viewing angles, FOV, and lens distortion still exist between the images acquired by the two cameras. To achieve pixel-level registration between the “multispectral input” and “hyperspectral label,” we employed a feature-point-based image registration method. The core workflow includes feature-point matching, geometric transformation model estimation, and FOV cropping, with the detailed procedure illustrated in Figure 5b.

First, the 9-channel multispectral data cube and the 127-channel hyperspectral data cube are averaged along the spectral dimension, respectively, to generate two representative grayscale images, which are then normalized to the grayscale range of 0–255 to serve as the inputs for feature matching. In the feature point detection and matching stage, the scale invariant feature transform (SIFT) algorithm [31] is employed to extract keypoints and compute feature descriptors, as this algorithm demonstrates good robustness against scale and rotation variations. Subsequently, the brute-force matching method is used for initial matching, followed by the application of Lowe’s ratio test to filter reliable matching points. The distance ratio threshold is set to 0.75 to balance the quantity and quality of the matches.

Based on the filtered matching point pairs, a Homography Transformation model is employed to describe the global geometric relationship between the two images. This model is characterized by a homography matrix M, representing translation, rotation, scaling, and perspective distortion. To robustly estimate M and exclude mismatches, the Random Sample Consensus (RANSAC) algorithm [32,33] is used for fitting, with a reprojection error threshold set to 1.0 pixels.

After obtaining the homography matrix M from the multispectral image to the hyperspectral image, the mapping positions of the four corner points of the multispectral image in the hyperspectral image coordinate system are calculated to determine its minimum bounding rectangle. Based on this, the hyperspectral data cube is cropped to obtain the corresponding FOV region. To achieve final pixel alignment, the cropped hyperspectral data cube is mapped back to the multispectral image coordinate system via the inverse transformation ***M***^−1^, ensuring that both datasets share identical spatial dimensions and pixel correspondence. This process yields spatially precisely registered pairs of 9-channel multispectral and 127-channel hyperspectral image cubes. Finally, spectral vector pairs are extracted pixel by pixel from each co-registered cube pair to form the dataset $\left\{I_{k},X_{k}\right\}$ for subsequent spectral reconstruction, where $I_{k}$ and $X_{k}$ represent the multispectral input and hyperspectral label, respectively.

### 2.4. Spectral Reconstruction

#### 2.4.1. Mathematical Formulation of Spectral Reconstruction

Following the image registration between the sub-channels of the divided-aperture system as described in Section 2.2, the multispectral data cube acquired by the 9-DAS MCam can be expressed as $i(x,y,\lambda_{p})$, where (x,y) denotes the spatial coordinate indices, $\lambda_{p}$ represents the p-th spectral channel (with p = 1, 2, …, n, here n = 9), and i corresponds to the grayscale digital values output by the detector. The hyperspectral data cube collected by the FTIR HCam can be expressed as $x(x,y,\lambda_{q})$, where $\lambda_{q}$ denotes the q-th spectral channel (q = 1, 2, …, m, here m = 127), covering a spectral range from 8 μm to 12 μm, and x represents the spectral radiance.

Through the dataset construction detailed in Section 2.3, the two data cubes mentioned above have achieved precise spatial alignment at coordinates (x, y), with each pixel corresponding to the same scene point. Therefore, the spectral reconstruction task can be formulated as follows: for each individual pixel, the corresponding 127-channel hyperspectral radiance $x_{q}$ must be estimated from its 9-channel multispectral observation $i_{p}$. This is equivalent to establishing a mapping function from the low-dimensional spectral vector I to the high-dimensional spectral vector X.

The spectral reconstruction process can be abstracted into the following model. For a specific pixel within the data cube acquired by the 9-DAS MCam, its measured grayscale value $i_{p}$ at the p-th spectral channel can be expressed as:

(7) $$i_{p}=L\left(\int_{\lambda_{min}}^{\lambda_{max}}h_{p}\left(\lambda \right)x\left(\lambda \right)d\lambda +n_{p}\right)$$

where $\lambda_{min}$ and $\lambda_{max}$ are the lower and upper limits of the operational wavelength range of the 9-DAS MCam, $h_{p}\left(\lambda \right)$ is the spectral response function of the p-th channel of the 9-DAS MCam, $x\left(\lambda \right)$ is the true spectral radiance of the target, provided by the FTIR HCam measurement results, $n_{p}$ represents noise, and L(⋅) denotes the detector’s analog-to-digital conversion relationship, which can typically be approximated as a linear function.

The spectral response function $h_{p}\left(\lambda \right)$ can be expressed as:

(8) $$h_{p}\left(\lambda \right)=l\left(\lambda \right)r\left(\lambda \right)f_{p}\left(\lambda \right)$$

where $l\left(\lambda \right)$ is the spectral transmittance of the lens assembly in the optical system, r(λ) is the spectral responsivity of the detector, and $f_{p}(\lambda )$ is the spectral transmittance of the filter corresponding to the p-th channel.

To facilitate numerical computation, the continuous model described above is discretized into a linear equation:

(9) $$i_{p}=L\left(\sum_{j=1}^{m}h_{pq}x_{q}\Delta \lambda +n\right)$$

Here, m denotes the number of spectral points used for discretizing the continuous spectrum, Δλ represents the spectral discretization interval, $h_{pq}$ denotes the system’s response function at the p-th channel and the q-th discrete spectral point, and $x_{q}$ indicates the spectral radiance of the target at the q-th discrete spectral point.

For a single pixel, the measured values from all nine channels can be represented as a vector $I\in R^{n\times 1}$, the high-resolution spectrum as a vector $X\in R^{m\times 1}$, and the system response as a matrix $H\in R^{n\times m}$, with noise denoted as $N\in R^{n\times 1}$. Consequently, the linear measurement model in matrix form is obtained as:

(10) $$I=L\left(HX+N\right)$$

The objective of this study is to reconstruct the high-dimensional spectrum X from the low-dimensional observation I, i.e., to solve the mapping:

(11) $$\hat{X}=F\left(I\right)$$

Here, $\hat{X}$ denotes the reconstructed estimate of X. Since the number of channels, n, is much smaller than the spectral dimension, m, this equation represents an underdetermined problem with infinitely many solutions. Therefore, we leverage the dataset $\left\{I_{k},X_{k}\right\}$ constructed previously to learn the underlying mapping function F(⋅) in a data-driven manner from a large number of samples. It is noteworthy that, as illustrated in Figure 2c, the filter array employed by the 9-DAS MCam provides uniform coverage with partial overlap across the 6–12 μm spectral range. This design ensures that no information is lost within the target wavelength region of 8–12 μm, thereby establishing the essential physical information foundation for reconstructing the hyperspectral signal X from the multispectral observation I.

#### 2.4.2. Network-Based Spectral Reconstruction Algorithm

The solution to Equation (11) can be formulated as the following optimization task:

(12) $$arg\;{\underset{\theta }{\min}\left\|X-\hat{X}\right\|}_{2}^{2}$$

That is, by optimizing the parameters θ of the algorithm, the reconstructed spectrum $\hat{X}$ is made to approximate the true hyperspectral signal X as closely as possible. Traditional spectral reconstruction methods often employ numerical approaches such as non-negative least squares [34], Tikhonov regularization [35,36,37], or compressed sensing [38]. While these methods offer strong interpretability, their reconstruction performance is limited in severely underdetermined scenarios where the number of channels n is much smaller than the spectral dimension m. In recent years, with the advancement of neural network technologies, data-driven spectral reconstruction methods have gradually demonstrated superior potential compared to traditional physics-constrained approaches [39].

In this study, the hyperspectral data cube acquired by the FTIR HCam retains only the lower-noise 8–12 μm spectral band, while the transmittance range of the lens and filters of the 9-DAS MCam is 6.5–12 μm. This results in a slightly broader spectral coverage for the 9-DAS MCam compared to the former, meaning that the first two channels of the 9-DAS MCam do not fully correspond to the 8–12 μm band. To address this spectral range mismatch, this paper adopts a purely data-driven reconstruction strategy. This method is trained on a large-scale paired dataset, enabling the network to adaptively suppress the weights of the first two channels of the 9-DAS MCam, thereby achieving effective spectral reconstruction.

**Figure 6:**
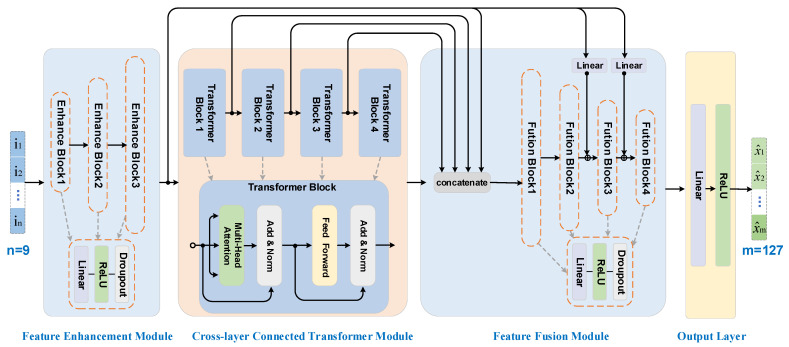
Schematic diagram of the overall architecture of the CTF-SRNet spectral reconstruction network.

Herein, this study proposes a Cross-layer Transformer Fusion for Spectral Reconstruction Network (CTF-SRNet). Its overall architecture, shown in Figure 6, primarily consists of a feature enhancement module, a cross-layer connected Transformer module, a feature fusion module, and an output layer.

First, the input multispectral vector is mapped into a higher-dimensional space through feature enhancement module. This module employs a three-layer fully connected structure, where each layer sequentially performs linear transformation, batch normalization, ReLU activation, and Dropout operations. This process embeds the low-dimensional input into a more expressive feature space to extract deep latent patterns.

The cross-layer connected Transformer module constitutes the core component of the model. In this module, stacked Transformer blocks employ the self-attention mechanism [40] to model global dependencies among spectral features, capturing long-range correlations across different spectral bands. To further aggregate features from varying depths, the module introduces cross-layer fusion pathways: the initial high-level features are concatenated along the feature dimension with the outputs from each Transformer sub-block, and then integrated into a unified representation through linear transformation. This mechanism facilitates the fusion of shallow, detailed information with deep, multi-level features, mitigating the loss of fine details in deep networks and enabling multi-scale feature integration.

The feature fusion module is responsible for progressively mapping the fused high-dimensional features to the target spectral dimension. This module consists of four fully connected layers, each comprising a linear transformation, batch normalization, and ReLU activation, with the feature dimension decreasing layer by layer. The first layer performs fusion and dimensionality reduction on the concatenated features. In subsequent layers, while receiving the output from the preceding layer, residual connections are employed to incorporate intermediate outputs from the feature enhancement module, thereby preserving detail integrity during reconstruction and alleviating the issue of gradient vanishing.

Finally, the output layer generates a non-negative 127-dimensional hyperspectral radiance vector through a linear layer followed by a ReLU activation function, ensuring the output adheres to physical constraints.

## 3. Results

### 3.1. Implementation of the 9-DAS MCam

**Figure 7:**
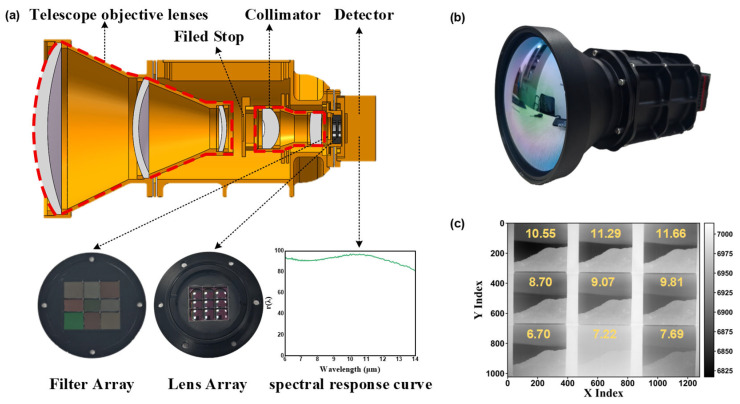
Hardware setup and raw output of the developed 9 DAS MCam. (**a**) Internal structure of the camera; (**b**) Photograph of the camera’s physical appearance; (**c**) Spatially tiled nine channel image directly output from a single camera exposure.

Based on the design outlined in Section 2.1, we have successfully developed the 9-DAS MCam. Figure 7a shows its internal structure. The system primarily consists of telescope objective lenses, a field stop, a collimator, the core spectral splitting assembly, and a infrared FPA detector. spectral splitting assembly includes a custom 3 × 3 bandpass filter array and a precisely aligned 3 × 3 lens array, which are key to achieving snapshot multispectral imaging. The external view of the camera is shown in Figure 7b.

Specifically, the 9-DAS MCam employs an optical system with a focal length of 50 mm and a F-number of 1.2, combined with a customized uncooled vanadium oxide infrared detector. The detector operates within the spectral response range of 6–14 μm, and its spectral response curve is shown in Figure 7a. It features a pixel array of 1280 × 1024 with a pixel pitch of 12 μm. Under the conditions of F/1.0, 30 Hz, and 300 K, its noise equivalent temperature difference (NETD) is less than 50 mK. The detector outputs 14-bit data at a frame rate of 30 Hz. The transmittance curve of the integrated 3 × 3 customized band-pass filter array is presented in Figure 2c.

The camera can simultaneously capture infrared images of a scene at nine specific spectral bands within a single exposure and output them in real time via a high-speed data interface, thereby validating its snapshot multispectral imaging capability. Figure 7c demonstrates a tiled nine-channel snapshot image of an outdoor scene captured by the system. It can be observed that the image from the channel with a central wavelength of 7.22 μm exhibits significantly higher overall brightness. This is primarily attributed to the fact that the full width at half maximum (FWHM) of the filter used in this channel (approximately 1 μm, see Figure 2c) is notably larger than that of the other channels (approximately 0.7 μm). The wider transmission band results in the reception of increased radiant energy, thereby leading to the higher image brightness.

### 3.2. Image Registration for Divided-Aperture Sub-Channels

**Figure 8:**
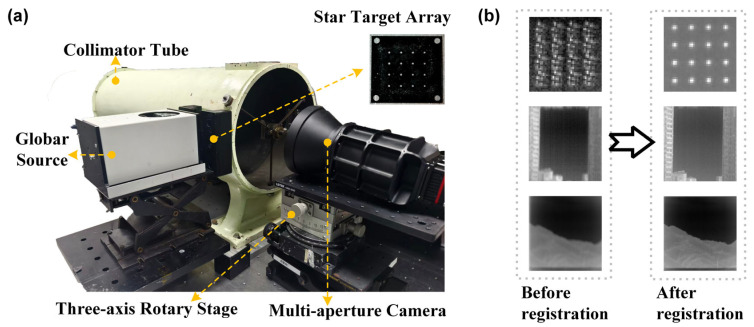
Experiment and results of divided-aperture sub-channel image registration. (**a**) Experimental setup for measuring inter-channel geometric deviations; (**b**) Comparison of image registration results before and after registration.

To achieve precise registration among the sub-channel images of the divided-aperture system, an inter-channel deviation calibration experiment based on the star-point array was conducted according to the method described in Section 2.2, as shown in Figure 8a. First, the theoretical aperture diameter of the star point was calculated using Equation (1), where  n = 1.5, the detector pixel size a = 12 µm, the collimator focal length $f^{\prime }$ = 2000 mm, and the imaging system focal length f = 49.7 mm. This calculation yielded a diameter of d = 725 µm. Based on this, a stainless-steel star-point array calibration plate with corresponding dimensions was designed and fabricated. During the experiment, the calibration plate was placed at the focal plane of the collimator tube. The camera was then moved via a three-axis translation stage to perform a FOV scan. After the camera captured star-point images covering the entire FOV, the grayscale-weighted centroid extraction method was applied to calculate the pixel position deviation of the star points in each channel relative to the central reference channel, thereby forming a deviation matrix.

Based on this deviation matrix, a geometric transformation model from each channel to the reference channel was established using a cubic polynomial, and image resampling was performed via bilinear interpolation to achieve precise registration of the sub-channel images. Figure 8b presents a comparative demonstration of the nine-channel superposition before (left) and after (right) registration, conducted in both laboratory and real outdoor environments. Under laboratory conditions, the star points from different channels were separated and exhibited noticeable ghosting before registration. After registration, the star points from all channels nearly completely overlapped, providing validation of the algorithm’s effectiveness.

To further evaluate its performance in real-world scenarios, imaging was performed on a building approximately 1000 m away and a mountain ridge approximately 3000 m away. Before registration, the superimposed images appeared blurred with indistinct contours due to inter-channel misalignment. After applying the proposed registration method, the textural details of the building became distinct, and the mountain ridge edges appeared sharp and clear, indicating a significant improvement in overall image quality. The experimental results demonstrate that the proposed method effectively corrects spatial deviations between channels in both laboratory and complex outdoor scenes, providing a pixel-aligned data foundation for subsequent hyperspectral reconstruction.

### 3.3. Training Data Acquisition

**Figure 9:**
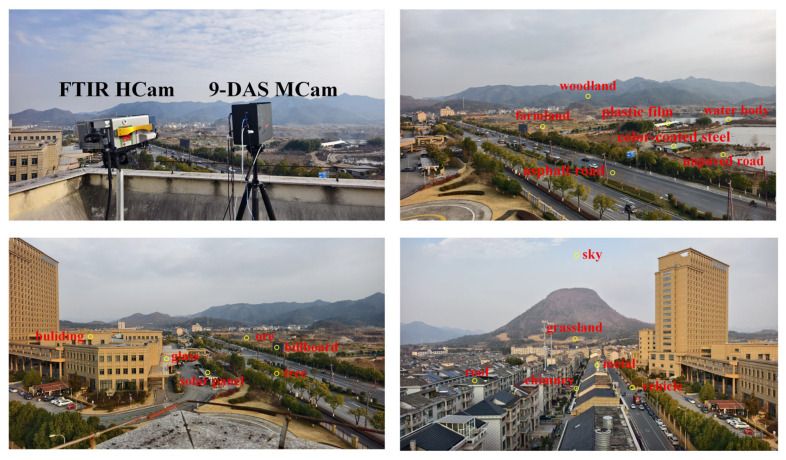
Outdoor acquisition scenario for the “multispectral-hyperspectral” paired dataset.

To train the spectral reconstruction neural network, this study constructed a paired dataset covering diverse scenarios. Data collection was conducted within Zhejiang Province, China, using synchronized acquisition with a 9-DAS MCam and an FTIR HCam. The captured scenes encompassed typical environments such as farmland, roads, water bodies, buildings, mineral sites, woodland, grassland, and various artificial materials (as shown in Figure 9), ensuring sufficient diversity in material composition and temperature distribution within the dataset.

Following the image registration process among divided-aperture channels described in Section 2.2, the multispectral data cube generated by the 9-DAS MCam has a spatial size of 218 × 223 pixels with 9 spectral channels, while the hyperspectral data cube acquired by the FTIR HCam has a spatial size of 320 × 256 pixels with 127 spectral channels.

According to Equation (4), the IFOV of the 9-DAS MCam is calculated as follows: given a detector pixel pitch of a = 12 μm and an effective focal length of f = 50 mm, the resulting *IFOV*_1_ is 0.24 mrad. Based on Equation (5), the IFOV of the FTIR HCam is determined by its FOV and pixel count: with a FOV = 6.4° × 5.1° and a pixel format of 320 × 256, the calculated *IFOV*_2_ is approximately 0.3485 mrad. Using Equation (6), the spatial scaling factor is derived as ρ ≈ 0.688. This indicates that, to maintain a consistent FOV, the imagery from the 9-DAS MCam must be spatially resampled to 68.8% of its original dimensions.

Based on the image registration method described in Section 2.3, the spectral data cube from the two cameras were aligned at the pixel level. After excluding cases with inadequate registration, a final dataset containing 1,292,800 paired samples of “multispectral input-hyperspectral label” $\left\{I_{k},X_{k}\right\}$ was constructed. This dataset was randomly split into training and testing sets in an 8:2 ratio for the subsequent training and evaluation of the spectral reconstruction task.

### 3.4. Spectral Reconstruction Results

**Figure 10:**
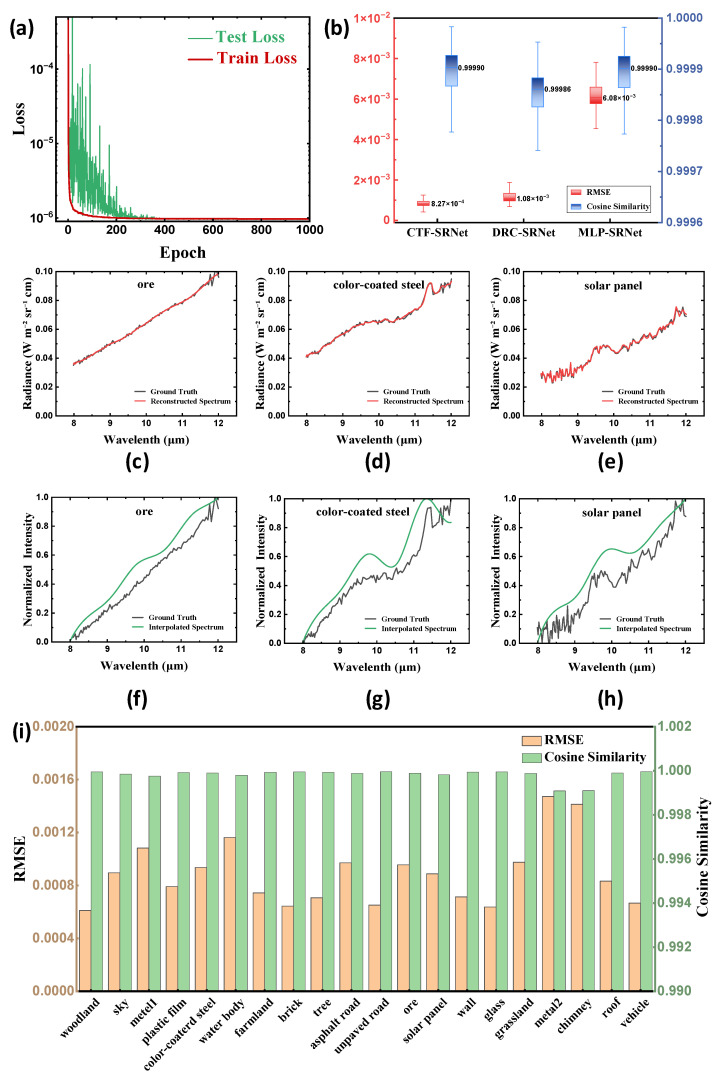
Evaluation of training convergence and reconstruction performance of the spectral reconstruction model. (**a**) Variation curves of training and testing loss versus training epochs; (**b**) Comparison of the statistical distribution of *RMSE* and $S_{cosine}$ for the reconstruction results of CTF-SRNet, DRC-SRNet, and MLP-SRNet on the test set; (**c**–**e**) Comparison between reconstructed spectral curves and ground truth measurements for three typical object targets; (**f**–**h**) Comparison between normalized intensity for multi-spectral interpolation results and ground truth measurements for three typical object targets. (**i**) RMSE and $S_{cosine}$ for 20 representative object categories.

The CTF-SRNet was trained on the training set, and its performance was validated on the validation set. As shown in Figure 10a, when the number of training epochs was set to 1000, both the training loss and validation loss converged, with no evidence of overfitting observed.

To quantitatively evaluate the quality of the reconstructed spectra, this study employs the Root Mean Square Error (RMSE) and the Cosine Similarity ($S_{cosine}$) as the evaluation metrics. These are used to assess the amplitude accuracy and the spectral shape consistency of the reconstructed spectra, respectively.

The RMSE measures the overall amplitude deviation between the reconstructed spectrum and the ground-truth spectrum, and its calculation formula is as follows:

(13) $$RMSE=\sqrt{\frac{1}{m}{\left\|X_{k}-{\hat{X}}_{k}\right\|}_{2}^{2}}$$

where m represents the number of spectral points. $X_{k}$ and ${\hat{X}}_{k}$ denote the ground-truth and reconstructed spectral vectors, respectively. A value of RMSE closer to 0 indicates higher accuracy in amplitude reconstruction.

Cosine similarity serves as a metric for spectral shape consistency, reflecting the degree of agreement in shape between two spectral vectors. It is calculated using the following formula:

(14) $$S_{cosine}=\frac{X_{k}\cdot {\hat{X}}_{k}}{\left\|X_{k}\right\|\cdot \left\|{\hat{X}}_{k}\right\|}$$

A value of $S_{cosine}$ closer to 1 indicates a greater similarity in shape between the reconstructed spectrum and the ground-truth spectrum.

The evaluation results on the test set are shown in Figure 10b. The RMSE was consistently maintained below 0.0015, close to zero, while the $S_{cosine}$ primarily ranged between 0.9997 and 1. This indicates that the model exhibits strong reconstruction performance.

To further evaluate the performance of the proposed CTF-SRNet, we trained two neural networks—the Multilayer Perceptron Spectrum Reconstruction Network (MLP-SRNet) and the Deep Residual Connection Spectrum Reconstruction Network (DRC-SRNet)—on the same dataset and obtained fully converged models. A performance comparison on the test set is shown in Figure 10b. It can be observed that the proposed model achieves better performance metrics, particularly in terms of RMSE, compared to both MLP-SRNet and DRC-SRNet, demonstrating its stronger capability in spectral reconstruction.

To demonstrate the reconstruction performance of the model, the trained model was used to reconstruct 20 spectral curves corresponding to 20 typical scene objects. Figure 10c–e present comparisons between the ground-truth and reconstructed spectra for three representative object types—ore (with no distinct spectral features), color-coated steel plates (with simple spectral features), and solar panels (with more complex spectral features), respectively. Figure 10i summarizes the distribution of RMSE and $S_{cosine}$ values for all 20 spectral curves, further validating the effectiveness and stability of the model across different spectral characteristics.

To validate the advantage of spectrally reconstructed data over raw multispectral data in material identification, we also applied cubic spline interpolation to the raw multispectral data of three materials—ore, color-coated steel, and solar panel—to simulate their continuous spectra. For better comparison, the interpolated data and the hyperspectral reference data acquired using the FTIR Hcam were normalized by mapping the maximum value to 1 and the minimum to 0. The results are shown in Figure 10f–h. The interpolated spectral curves can only reflect the overall spectral profile, failing to capture detailed spectral features. In particular, the variation trends of the interpolated curves for color-coated steel and solar panel are quite similar. Without knowledge of their fine spectral characteristics, relying solely on low-resolution raw multispectral data can easily lead to misclassification between these two types of materials. This further highlights the necessity of reconstructing high-fidelity, high-resolution spectra from multispectral data.

**Figure 11:**
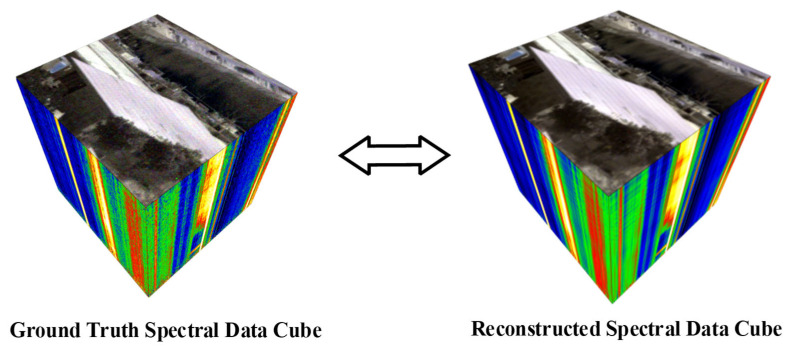
Comparison of spectral data cubes, visualized through false-color composite: ground-truth (**left**) versus reconstructed (**right**).

Finally, to visually demonstrate the model’s overall reconstruction performance in the spatial-spectral domain, a hyperspectral image (size 225 × 236) was reconstructed using the proposed CTF-SRNet method on a platform equipped with an AMD Ryzen 7 5800X 8-core processor and an NVIDIA GeForce RTX 3060 Ti GPU, with a reconstruction time of 3.36 s. Figure 11 presents a comparative display of the ground-truth spectral data cube acquired by the FTIR HCam and the spectral data cube reconstructed by the model. It can be observed that the reconstructed data cube shows a high degree of consistency with the ground truth in terms of spectral features, indicating that the model effectively learns and recovers the spectral information for each pixel. Furthermore, the reconstructed data cube exhibits a lower noise level visually, suggesting that the model possesses a certain noise suppression capability while accurately reconstructing the spectra.

## 4. Discussion

This study proposes a imaging method that integrates a divided-aperture snapshot hardware system with data-driven computational methods, and preliminarily validates its feasibility for achieving simultaneous snapshot imaging and hyperspectral information acquisition in the thermal infrared band. Compared to traditional spectral imaging methods, the core advantage of the proposed system lies in its simple, motionless divided-aperture spectral imaging mechanism, which enables the acquisition of hyperspectral information within a single exposure. This capability offers the potential to capture the spectral-temporal evolution of dynamic processes such as combustion or gas leakage. Experimental results demonstrate that, despite the hardware supporting only 9 spectral channels, the data reconstruction method based on deep neural networks can fully exploit physical correlations and statistical priors among spectral bands, achieving high-quality reconstruction targeting 127 spectral bands. This showcases the significant potential of computational imaging techniques to overcome hardware limitations.

However, this method currently has several limitations. First, the reconstruction performance heavily relies on the quality and coverage of the paired training data. Although multi-scene data have been collected for training, the dataset may still lack samples of extreme-temperature targets or materials with highly distinctive spectral features, which could affect the model’s generalization capability when encountering unknown or anomalous spectral distributions. Furthermore, this study primarily validates the feasibility of the technical approach. To achieve reliable monitoring in practical scenarios, it is still necessary to utilize hyperspectral calibration equipment (such as an FTIR HCam) to synchronously collect data under different time frames and environmental conditions, thereby training a snapshot hyperspectral imaging system with practical applicability.

On the other hand, although the system has achieved snapshot imaging, extending pixel-level hyperspectral reconstruction to video-rate applications will face significant data processing demands, requiring highly parallel computational architectures to enable real-time processing. It is worth noting that in many monitoring tasks, acquiring full-FOV hyperspectral data throughout the entire process is unnecessary. Instead, anomaly detection can first be performed based on the 9-channel images, followed by localized hyperspectral reconstruction specifically for regions of interest. This approach can substantially reduce computational load and enhance the system’s operational practicality.

Furthermore, constrained by current research conditions, this study employs a purely data-constrained optimization method for spectral reconstruction. Future work could involve deeper integration of the reconstruction network with the physical model of the imaging system to enhance the interpretability of the reconstruction process and the stability of the results. In summary, this research provides a viable technical pathway toward real-time, compact thermal infrared hyperspectral imaging. Its core concepts can also be extended to other optical imaging fields where high spatio-temporal and spectral resolution are urgently required.

## 5. Conclusions

This study designed and implemented a thermal infrared hyperspectral computational imaging method based on a snapshot divided-aperture system. By developing the 9-DAS MCam, the parallel acquisition of multispectral information within a single exposure was achieved at the hardware level. To address the inherent inter-channel spatial deviations of the divided-aperture system, a precise registration method based on a star-point array was proposed, effectively realizing pixel-level spectral alignment. Building on this, a paired dataset synchronously acquired with a FTIR HCam was constructed. Furthermore, a CTF-SRNet was designed, successfully reconstructing 127-channel hyperspectral data from nine low-dimensional spectral channels.

Experimental results demonstrate that this integrated solution achieves high-precision spectral recovery while maintaining a compact system structure and video-rate temporal resolution. This provides a practical new pathway to resolve the traditional conflict between “snapshot imaging capability” and “high spectral resolution” for dynamic scenes in the thermal infrared band, holding significant application potential in fields such as environmental monitoring, industrial inspection, and security reconnaissance.

## Data Availability

The data presented in this study are available on request from the corresponding authors.

## Abbreviations

The following abbreviations are used in this manuscript:

9-DAS MCam	Nine-channel divided-aperture snapshot multispectral camera
FTIR HCam	Fourier-transform infrared hyperspectral camera
CTF-SRNet	Cross-layer Transformer Fusion for Spectral Reconstruction Network
FPA	Focal plane array
FOV	Field of view
IFOV	Instantaneous field of view
SNR	Signal-to-noise ratio
SIFT	Scale invariant feature transform
RANSAC	Random Sample Consensus
FWHM	Full width at half maximum
